# Comparative genomics reveals substantial divergence in metal sensitive and metal tolerant isolates of the ericoid mycorrhizal fungus *Oidiodendron maius*

**DOI:** 10.1007/s00572-025-01191-x

**Published:** 2025-03-21

**Authors:** Stefania Daghino, Claude Murat, Stéphane De Mita, Elena Martino, Silvia Perotto

**Affiliations:** 1 Institute for Sustainable Plant Protection, CNR, Strada Delle Cacce 73, 10135 Turin, Italy; 2 Université de Lorraine, INRAE, UMR Interactions Arbres/Microorganismes, Centre INRAE Grand Est Nancy, Champenoux, France; 3 INRAE, CIRAD, PHIM, Univ Montpellier, Institut Agro, IRD, Montpellier, France; 4 Department of Life Sciences and Systems Biology, University of Torino, V. le Mattioli 25, 10125 Turin, Italy

**Keywords:** Mycorrhizal fungi, *Oidiodendron*, Metals, Comparative genomics, Polymorphism

## Abstract

Some heavy metal tolerant fungal isolates capable of forming ericoid mycorrhiza can also confer increased metal tolerance to the host plant. One of these fungal isolates, *Oidiodendron maius* Zn, has been characterized and a few molecular mechanisms underlying its metal tolerant phenotype have been identified. Here, we investigate the genomic divergences between the available genome of *O. maius* Zn and the genomes of metal tolerant and sensitive isolates of *O. maius*, with the aim of identifying genes or intergenic regions possibly involved in the display of the tolerance. The resequenced genomes of 8 tolerant and 10 sensitive isolates were mapped on the reference, *O. maius *Zn, yielding 357 gene models from the reference that were either missing or too polymorphic to be identified in the genomes of the sensitive isolates. These regions included genes with functions related to defense mechanisms and with unknown functions. One third of the predicted gene models turned out to be highly polymorphic, including many enriched GO terms, i.e. DNA/RNA metabolism and modification, chromosome/chromatin organization, protein biosynthesis, metabolism and function, energy consumption/transfer and mitochondrion. Overall, our findings indicate that the tolerant phenotype in *O. maius* likely arises from multiple genetic adaptations rather than a singular mechanism.

**Supplementary Information:**

The online version contains supplementary material available at 10.1007/s00572-025-01191-x.

## Introduction

Ericoid mycorrhiza (ErM) involves fungal symbionts mainly in the Hyaloscyphoid clade of the order Helotiales, class Leotiomycetes (Ascomycetes) and in the Serendipitaceae family, order Sebacinales (Basidiomycetes), and the fine hair roots of plants of the Ericaceae family, such as blueberry (*Vaccinium* sp.), heather (*Calluna* and *Erica* sp.), and rhododendron (*Rhododendron* sp.). Densely intertwined intracellular fungal coils are formed inside ericaceous plant hair root epidermal cells, with fungal mycelium coming directly from soil or from neighboring rhizodermal cells; hyphal sheaths on the hair root surface sometimes occur (Vohnik, 2020). Ericaceous plants dominate in nutrient-poor, generally acidic soils, rich in recalcitrant compounds and characterized by a slow rate of soil organic matter (SOM) decomposition. The low pH and anaerobic soil conditions facilitate mobilization of heavy metal ions (Meharg and Cairney 2000). Ericoid fungi have maintained a rich repertoire of genes encoding for hydrolytic enzymes (Martino et al. 2018), and this saprotrophic ability enables them to mineralize complex SOM, thus providing simple P and N forms to the ErM host plant. Moreover, ErM fungi greatly increases metal tolerance of the host plant in such heavy metal enriched soils (Bradley et al. 1981, 1982), although the mechanisms involved remain largely unknown (Casarrubia et al. 2020). A metal tolerant phenotype has been described for many ErM fungi isolated from sites with different levels and kinds of pollution (Martino et al. 2000a; Sharples et al. 2000; Vallino et al. 2011). However, our understanding of the mechanisms underlying metal tolerance of ErM fungi is limited to a few mechanisms identified in the metal-tolerant strain *O. maius* Zn, isolated from an experimental plot in the Niepolomice Forest (Poland) heavily contaminated with industrial dusts and containing high concentrations of Zn, Cd, and Al (Greszta et al. 1987). Although the archetypal ErM fungal species, *Hyaloscypha hepaticicola,* belong to the *H. hepaticicola* aggregate (previously known as *Rhizoscyphus ericae* aggregate or *Hymenoscyphus ericae* aggregate), also including *H. gryndleri*, *H. finlandica*, *H. variabilis* and *H. bicolor*, also *O. maius*, was later attributed to this group of quite diverse symbiotic fungi (Vohnik, 2020; Bruzone et al. 2015, Vohnik et al. 2023), with some other ascomycetes forming hyphal coils in the roots of ericaceous plants in vitro (e.g. *Acremonium strictum, Geomyces pannorum*) and some Dark Septate Endophytes (DSE) of the *Phialocephala fortinii* s. l.—*Acephala applanata* species complex forming loose intracellular hyphal loops similar to ericoid coils (Vohink, 2020).

Among *O. maius* Zn (hereafter called OmZn) tolerance mechanisms, antioxidant responses, mediated by superoxide dismutase (Vallino et al. 2005, 2009; Abbà et al. 2009), metal transport and compartmentalization mediated by two metal transporters (Khouja et al. 2013), nitrogen and carbon metabolism mediated by extra- and intracellular enzymes (Khouja et al. 2014; Martino et al. 2000b), solubilization mediated by low molecular weight organic acids (Martino et al. 2003) and DNA repair mechanisms (Abbà et al. 2011; Daghino et al. 2019) have been described. However, the genetic traits determining metal tolerance of ErM fungi are still poorly understood.

Previous experiments involving some *O. maius* isolates suggested a higher rate of DNA polymorphism in the promoter region of the Cu/Zn superoxide-dismutase gene in fungal isolates from heavily polluted sites than in those from unpolluted or mildly polluted sites (Vallino et al. 2011). In addition, the comparison of a Ni/Cr-sensitive isolate of *O. maius* with a tolerant isolate revealed the lack of a glutathione synthase gene in the former (Murat et al. 2011). Since glutathione is known to be involved in metal tolerance, the authors speculated that this genetic difference may explain the phenotypic difference, although the need for further experiments was suggested (Murat et al. 2011). Overall, these previous studies, based both on targeted and on small scale untargeted approaches, contributed to the discovery of genes involved in the heavy metal tolerant phenotype of OmZn. Casarrubia et al. 2020 measured a reduced Cd content in *Vaccinium myrtillus* roots colonized by OmZn exposed to this metal, if compared to non-mycorrhizal roots. The release of the complete genome sequence of OmZn (Kohler et al. 2015) allowed these authors to perform transcriptomic investigations in OmZn free living mycelium and in symbiosis in response to Cd. They highlighted the down-regulation in symbiosis of metal transporters known to transport Cd, possibly explaining the reduced Cd content recorded in mycorrhizal roots. However, this transcriptomic study focused on the differential expression of genes somehow expected to be involved in metal tolerance (e.g., metal transporters), whereas a genome-scale approach with no a priori candidate gene or function to search for genes involved in metal tolerance has not yet been accomplished for ErM fungi. The scan for genomic polymorphisms among groups of individuals with different phenotypes has the potential to identify the most polymorphic regions and, within them, possible candidate genes linked to distinctive phenotypic traits, such as virulence or adaptation to different hosts or environments (Schmidt et al. 2016). Intraspecific genetic changes are reported to rely mainly on non-synonymous mutations (i.e. leading to amino acid substitutions), gene regulation and gene gains, losses and duplications (Gladieux et al. 2014). The analysis of genetic variation among isolates of the poplar rust fungus *Melampsora larici-populina*, characterized by distinct virulence profiles, indicated a correlation between non-synonymous SNPs in genes encoding secreted proteins and the virulent phenotype (Persoons et al. 2014), and led to the identification of a single gene which may have allowed an adaptive event in virulence (Persoons et al. 2022). A similar approach was used to investigate adaptation of *Suillus brevipes* to salinity, by resequencing 28 isolates from a seaside and from a montane site. With this approach, the authors identified a genomic region with extreme divergence containing a gene encoding for a membrane Na^+^/H^+^ exchanger known to enhance salt tolerance in plants and yeast (Branco et al. 2015), further supporting the use of polymorphism scan of different genomes to identify putatively adaptive genes. More recently, Bazzicalupo et al. (2020) analyzed the genomes of *Suillus luteus* isolates from heavy metal polluted or non-polluted soils, looking for genomic traces of adaptation. Although they found an overall low divergence across the isolates, genes involved in metal homeostasis, storage, immobilization and ROS detoxification strategies were identified within the most diverging genome regions, suggesting that a polygenic adaptation to heavy metal pollution might be ongoing. An experiment of adaptive laboratory evolution resulted in the adaptation of *Pleurotus ostreatus* to an extreme Cd gradient and led to the onset of Cd-tolerant mutants with over 2,500 SNPs, 70 indels and 39 copy number variation, with mutant genes being primarily involved in oxidation–reduction reactions, ion transmembrane transport and metal compartment/efflux genes (Wang et al. 2024). The aim of our study was to investigate phenotypic and genotypic divergence of *O. maius* isolates, in order to identify genomic regions and gene models (i.e. a region of the genome transcribed into RNA which is then either translated into protein, or it belongs to one of a number of defined classes of noncoding RNA genes—Schnable 2019) that may potentially explain metal tolerance in some of these isolates. For this purpose, 8 metal tolerant and 10 metal sensitive isolates of *O. maius*, used in previous studies where preliminary growth assays in metal-amended culture media have been performed (Martino et al. 2000b, 2003; Lacourt et al. 2000; Vallino et al. 2011), were further characterized for their phenotype in vitro and their genomes were sequenced and compared with the reference genome of OmZn. Metal-tolerant and metal-sensitive isolates were sampled from both sympatric and allopatric locations, reducing the risk of confusion between adaptation toward metal tolerance and differentiation caused by population divergence. The hypothesis was to find some highly diverging regions/gene models, but we found that the divergence involved one third of the gene models, providing a picture of wide genetic changes probably at the base of OmZn metal tolerance.

## Methods

### Fungal isolates and culture conditions

The 18 *O. maius* isolates used in this paper are listed in Table 1S. The fungal isolates are deposited in the Mycotheca Universitatis Taurinensis (MUT, University of Turin, Italy). They were isolated from roots of plants collected in four different sites, either unpolluted (San Francesco al Campo, Italy; Canada) or with soils naturally containing (Mt Avic Park, Col de la Croix, Italy) or artificially amended with (Niepolomice forest, Poland) heavy metals. The fungi were grown on liquid Czapek Dox mineral medium (Oxoid, pH 6), and the mycelia were filtered after 30 days and stored at −80° C for the gDNA extraction. In order to assess growth of the *O. maius* isolates exposed to heavy metals, a solid Czapek-glucose mineral medium (glucose 2% w/v, 35 mM NaNO_3_, 5.7 mM K_2_HPO_4_٠ 3H_2_O, 2 mM MgSO_4_٠7H_2_O, 6.7 mM KCl, 36 μM FeSO_4_٠7H_2_O, 20 mM MES hydrate, agar 1%, pH 5.6; all reagents from Sigma) was prepared without metals (control) or amended alternatively with ZnSO_4_٠7H_2_O 15 mM (hereafter called Zn), with 3CdSO_4_٠8H_2_O 0.3 mM (hereafter called Cd), with K_2_Cr_2_O_7_ 0.6 mM (hereafter called Cr) or with NiSO_4_٠7H_2_O 0.6 mM (hereafter called Ni). These concentrations have been selected based on literature data (Martino et al. 2000a; b; Lacourt et al. 2000; Vallino et al. 2011). Sterile cellophane membranes were placed on the agar surface before fungal inoculation, to provide a convenient means of collecting the mycelium from the plate at the end of the experiment. To get rid of chemicals added for conservation, the membranes were first boiled for 15 min in 10 mM EDTA-Na_2_ (Sigma), rinsed and then autoclaved in ddH_2_O. Fungal mycelia were collected from the plates after a 30 days incubation at 25 °C and their dry biomass was recorded. A principal component analysis (PCA) was performed on the biomass data by using tools provided in R (v. 4.1.0), such as res.pca to compute the PCA and fviz_pca_biplot to visualize the biplot of individuals and variables, both available from the R package factoextra (v. 1.0.7).

In order to distinguish between sensitive and tolerant isolates, a tolerance index (TI) was calculated as the ratio between the biomass dry weight of the mycelium exposed to the metal and the biomass dry weight of the mycelium grown in control conditions (Colpaert et al. 2004). We refer to isolates as tolerant when the TI falls over the 90th percentile of the distribution of the average TI in the presence of one of the tested metals.

### Genomic DNA extraction

The frozen mycelia were finely ground in liquid nitrogen, the genomic DNA was extracted following two slightly different protocols, and the best extract, based on integrity and purity, was used for sequencing. In the first protocol, gDNA was extracted in a sorbitol extraction buffer (Buffer A: 100 mM Tris–HCl, pH 9.0, 0.35 M sorbitol, 5 mM EDTA, pH 8.0; buffer B: 200 mM Tris–HCl, pH 9.0, 2 M NaCl, 2% CTAB, 50 mM EDTA, pH 8.0; buffer C: sarkosyl 5% aqueous solution; A:B:C = 2,5:2,5:1, PVP 0.1% and PVPP 2% added prior to use, incubate 1 h 65 °C), purified by phenol:chloroform:isoamyl alcohol and chloroform extraction (Kohler et al. 2015), RNase A treated (0.01 mg per 100 µl of extract, 15' at 37 °C), isopropanol precipitated and resuspended in TE (Tris HCl 10 mM pH 8, EDTA 1 mM). In the second protocol, gDNA was extracted in Tris–HCl extraction buffer (Tris–HCl 100 mM pH 8, NaCl 100 mM, Na-EDTA 20 mM, PVP 0.1%, Na-lauroylsarcosine 1% in H_2_O) purified by phenol:chloroform:isoamyl alcohol and chloroform extraction, isopropanol precipitated and resuspended in ddH_2_O, the RNA was precipitated with LiCl 3 M and the gDNA in the supernatant was precipitated again with isopropanol and resuspended in TE.

The DNA solutions obtained were further purified using Qiagen genomic-tip 100/G columns following the manufacturer’s instructions, precipitated with sodium acetate 3 M and isopropanol and resuspended in TE. The quality and quantity of the DNA solutions were checked by Nanodrop and Qubit, in order to verify that the samples meet the requirements for gDNA Illumina Sequencing.

### Phylogenetic analysis

The ITS2 regions of the nuclear rDNA, comprised between the 5.8S and the 28S rRNA genes, were amplified and sequenced using the generic eukaryotic primers ITS3 (GCATCGATGAAGAACGCAGC) and ITS4 (TCCTCCGCTTATTGATATGC) according to White et al. (1990). PCR amplicons were sequenced using the Sanger method (Macrogen). The chromatograms of the sequences obtained were verified visually using Seqtrace-0.9.0. *Oidiodendron* spp. sequences retrieved from Wei et al. (2016) have been included in the phylogenetic analysis, as well as the ITS sequence of the *O. maius* type strain from CBS 402.69. The multiple sequence alignment and the Neighbor Joining (NJ) analysis with Jukes-Cantor distances and 1000 bootstrap replicates were performed with MAFFT (version 7, Katoh et al. 2019). A *Pseudogymnoascus roseus* ITS sequence was used as an outgroup.

### DNA sequencing, mapping and variant calling

Genomic DNA libraries were prepared for all isolates using TruSeqNano kit followed by paired-end 100 base pairs sequencing on Illumina HiSEQ2000 performed by the Genomic Platform of the “Genopole Toulouse Midi-Pyrénées” (France). Adapter and quality filtering were carried out using Trimmomatic (Bolger et al. 2014). The raw-read data were deposited in the NCBI SRA sequence reads archive under Accession No PRJNA1101509.

Fastq files of trimmed sequences were used to proceed with mapping onto the available OmZn reference genome (JGI; http://genome.jgi.doe.gov/programs/fungi/index.jsf; Kohler et al. 2015) and variant calling. These two steps have been performed using two different methods: (i) CLC-method based on the Genomics Workbench 8.0 software (CLC bio, QIAgen, Aarhus, Denmark) and (ii) BWA-v0.7.12/SAMtools-v1.2 method (https://github.com/sdemita/o_maius_zn.git). Stringent parameters were used for mapping with both methods (98% of similarity of the sequence, 90% of the length of the sequence aligned). For method (i) the 100 scaffolds composing the reference genome were uploaded in CLC Genomics Workbench and the annotation was imported using the annotation plugin. The following parameters have been used for mapping: length fraction = 1.0, similarity fraction = 0.9, while the other parameters have been set to default. The SNPs were called by the quality-based variant detection module using default parameters (i.e. minimum coverage of 10 reads, minimum variant frequency 35%) and maximum expected ploidy set to 1 because the OmZn genome is haploid.

For method (ii) the raw reads for each genome were aligned to the reference genome using the Burrow-Wheeler aligner (BWA, v0.7.12; Li and Durbin 2009) according to Payen et al. (2015). Only reads mapped with a MAPQ calculated by BWA above 25 were considered for analysis with SAMtools (Li et al. 2009). The alignment output was used to create a pile-up file (i.e. file describing the mapping results information at each genome position) by the SAMtools mpileup command. The BCFtools (v1.2) within SAMtools were used for calling the genome variants that were further filtered for quality and depth by the vcfutils script (VCFv4.2). To be validated, each SNP was required to be supported by at least ten reads, and the root mean square (RMS) of the mapping quality of the SNP position had to be ≥ 30. The coverage of the mapping obtained with the BWA/SAMtools method was calculated using the coverage and genomecov tools of the BEDtools suite (2–2.27.1). Since *O. maius* mycelium is haploid, only the homokaryotic SNPs called with both methods were considered for further analyses and were annotated using SNPEff (v4.1 g, Cingolani et al. 2012b) and filtered with SNPSift (Cingolani et al. 2012a).

A neighbor joining phylogeny with all SNPs was realized using Quicktree (Howe et al. 2002), and 100 bootstraps. Phylogeny was visualized using FigTree (http://tree.bio.ed.ac.uk/software/figtree/).

### Divergence calculation

The level of polymorphism between the genomes of tolerant and sensitive isolates was assessed by calculating the F_st_ and Tajima’s D (D) values (Tajima, 1989) of each gene and of 10 Kb sliding windows using EggLib version 3.0.0b25 (Siol et al. 2022, https://github.com/sdemita/o_maius_zn.git). The F_st_ estimator usually ranges from 0 to 1, but it can also have negative values which do not have a biological meaning (Willing et al. 2012). We considered the genes and windows with F_st_ > 0.5 as highly divergent. In order to exclude gene models that display genetic differentiation due to geographic distribution rather than phenotype (i.e., tolerant versus sensitive to heavy metals), F_st_ values were calculated considering both the geographic origin and the phenotype. Only gene models with higher F_st_ value based on phenotype than geographic origin were considered. D values have an expected normal distribution between −2 and + 2 for a 95% confidence interval (Tajima 1989). Therefore, we considered in this study values higher than + 2 for balancing selection or lower than −2 for positive selection. In addition, in order to identify candidate genes among those with very high values of F_st_, we also calculated the statistical outlier of the F_st_ value distribution, according to the Tukey’s boxplot rules, based on the interquartile range calculation, IQR (Brandt 1990). Outliers are defined as those falling below (Q_1_ − 1.5) of the IQR or above (Q_3_ + 1.5) IQR, where Q_1_ is the lower quartile and Q_3_ is the upper quartile.

### Gene ontology and KOG enrichment analysis

The analysis of KOG (EuKaryotic Orthologous Groups) categories differently represented (Fisher test p-value < 0.05) between groups of genes that were either present/absent in the genomes, or highly/slightly polymorphic, have been performed using an on-line Fisher exact test calculator (https://www.socscistatistics.com/tests/fisher/default2.aspx).

A Gene Ontology enrichment analysis was performed on different groups of gene models selected either because they were not covered by the sequencing, or because of their relevance for the estimated polymorphism or fixation (see above). The ClueGO (2.5.9, Bindea et al. 2009) plug-in for Cytoscape (3.7.2, Shannon et al. 2003) was used, with the following selection criteria: Statistical Test Used = Enrichment; p value cutoff = 0.05; Correction Method Used = Bonferroni step down; Min GO Level = 3; Max GO Level = 8; Number of Genes = 3; Min Percentage = 4.0; GO Fusion = true; GO Group = true; Kappa Score Threshold = 0.5; Group By Kappa Statistics = true.

## Results

### Characterization of the *O. maius* isolates: phenotypic response to heavy metals

**Fig. 1:**
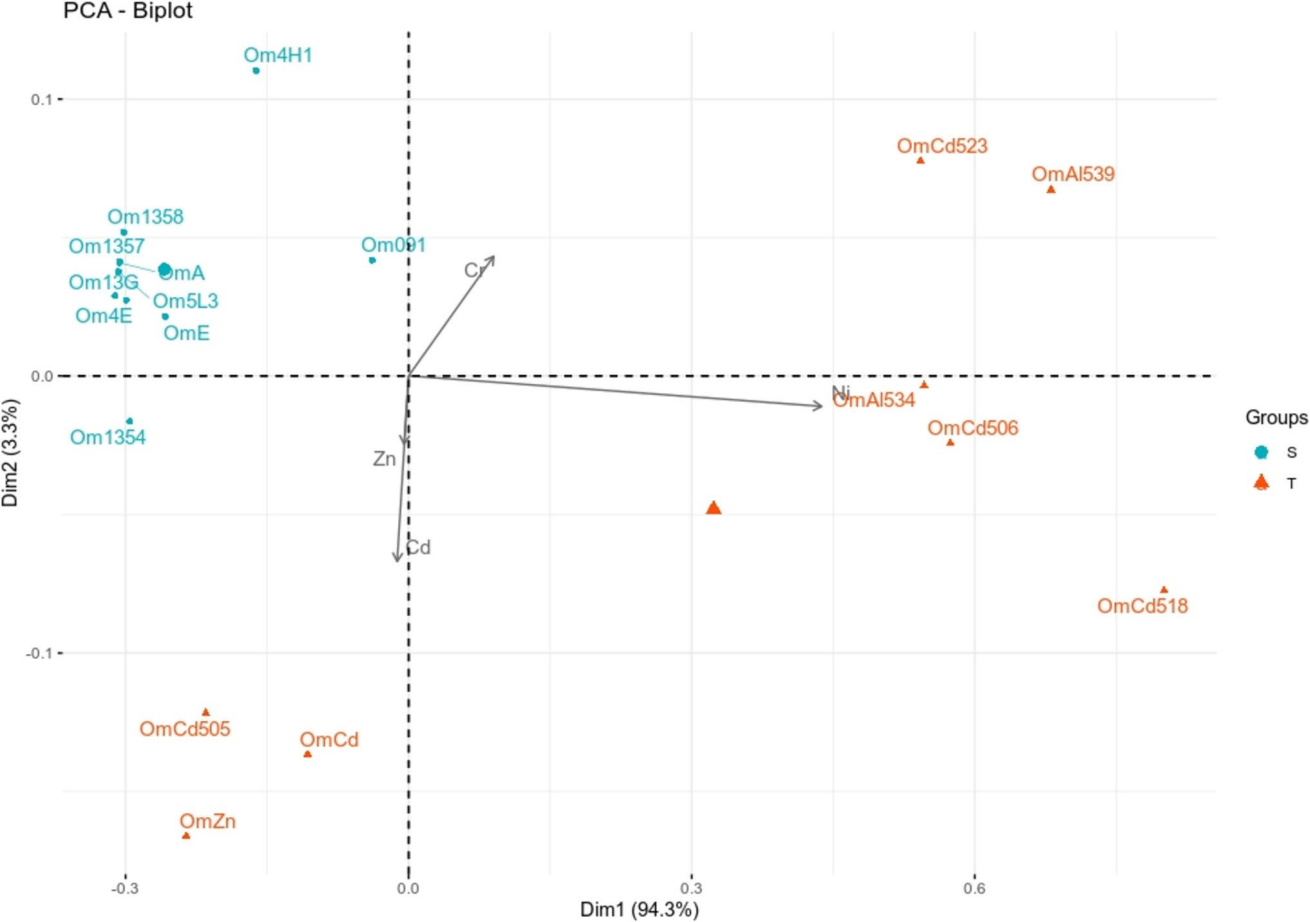
Principal component analysis performed on the isolates’ biomass data. The first dimension separates the isolates tolerant to Cr/Ni from the others, while the second dimension separates the Zn/Cd tolerant isolates from the others. The sensitive isolates, in light blue, are separated from the two above mentioned groups. The red triangle and the blue dot represent the mean point of the group of the sensitive and tolerant isolates, respectively

**Fig. 2:**
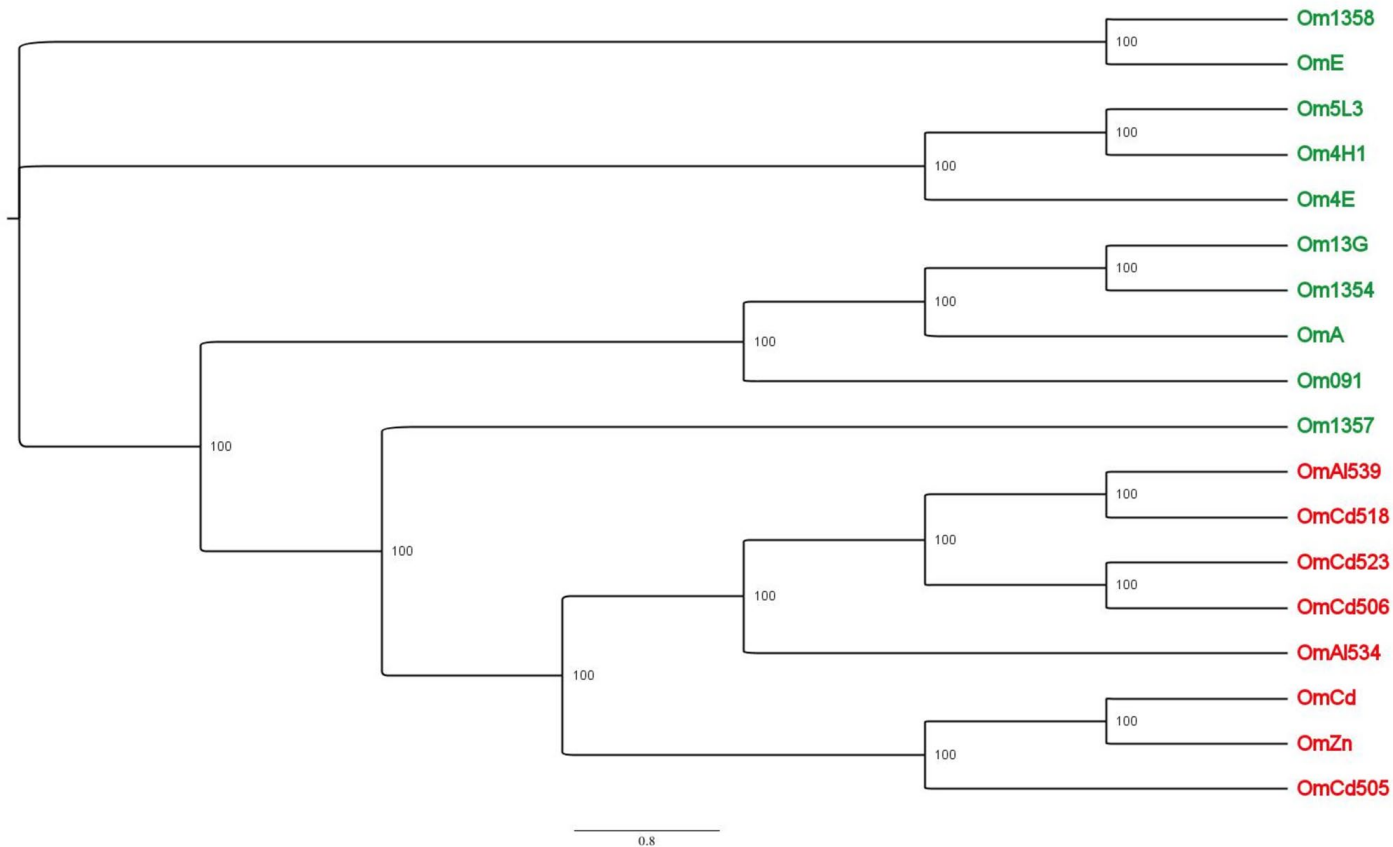
Phylogeny of the *O. maius* strains considered in this study based on the alignment of all the polymorphic positions in the full genome. The metal-sensitive isolates are in green, while the tolerant ones are in red (unrooted neighbor joining tree, with bootstrap values as node labels)

According to the phylogenetic analysis based on the ITS sequences, the 18 *O. maius* isolates clustered together with the *O. maius* type sequence (Fig. 1S). We tested the growth of these 18 *O. maius* isolates in media containing different metals. Some isolates were unable to grow in the presence of heavy metals, while others could grow in the presence of at least one metal. The yield of each isolate on the different metals was very different. For example, the top Ni-tolerant isolate (Om518) grew more on Ni-amended medium than in control conditions, while the top Cd-tolerant isolate (Om505) grew on Cd-amended medium up to 20% of the same isolate in control conditions. For this reason, we decided to consider as heavy metal tolerant those isolates with an average tolerance index (TI, see method section) that falls within the upper 10% of the TI of the analyzed isolates on that metal. This phenotype analysis allowed us to classify the isolates in two groups: 8 tolerant and 10 sensitive isolates. In particular, eight isolates were found to be tolerant to at least one metal (OmZn, OmCd, OmCd505, OmAl534, OmCd506, OmCd518, OnCd523, OmAl539), whereas 10 isolates were sensitive to all metals (Om5L3, OmA, OmE, Om091, Om13G, Om4E, Om1354, Om1357, Om1358, Om4H1) (Fig. 2S). Sensitive isolates clustered together in the principal component analysis (Fig. 1), whereas tolerant isolates were split in two subgroups according to the metal(s) they were tolerant to. Axis 1 discriminated isolates tolerant to Ni and/or Cr (OmAl534, OmCd506, OmCd518, OmCd523, OmAl539), while axis 2 discriminated those tolerant to Zn and/or Cd (OmZn, OmCd and Om505). No correspondence was found between ITS-based clustering and either heavy metal tolerance or geographic origin of the fungal isolates (Fig. 1S).

### Genome resequencing: reads mapping and gene models specific to metal tolerant and metal sensitive isolates

**Fig. 3:**
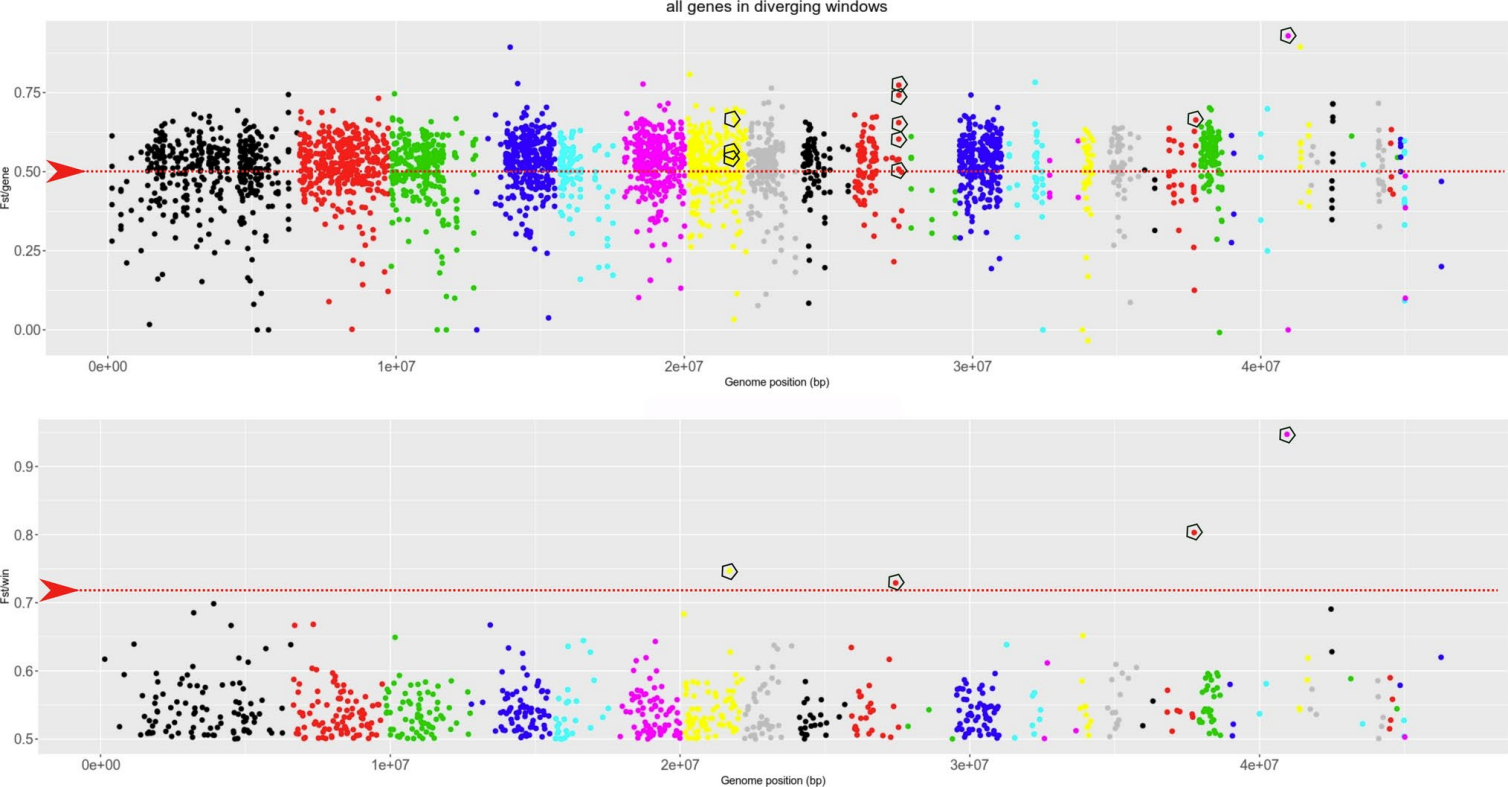
Diverging genes found in diverging genome regions. Upper panel: each point represents the F_st_ calculated for each gene harbored in a diverging window represented in the lower panel. The genes above the red dashed line are highly polymorphic (Fst > 0.5) and those indicated by pentagons fall in the F_st_-outlier windows. Lower panel: each point represents the F_st_ calculated for a 10 Kb genome window, with a minimum threshold of 0.5. The red dashed line indicates the value that separates the statistical outliers (see methods section), that are also indicated by a pentagon. Different colors indicate the position of the window/gene in a different genome scaffold

After filtering for quality, a total of 318 × 10^6^ reads were obtained, corresponding to 10 x 10^6^ to 33 × 10^6^ reads per isolate (Table 2S). On average, 55% (CLC) or 68% (BWA/SAMtools) of the reads aligned to the reference OmZn genome. The base coverage was between 73 and 99%, and the depth was always higher than 14X, with an average of 26X (Table 2S). Isolates OmCd and Om505 showed the highest read coverage, with reads from OmCd covering more than 99% of the reference genome, including all gene models. For the other isolates, a total of 920 genomic regions were not covered by reads, corresponding to 930 gene models of the reference genome (Table 3S-Sheet 1). For these gene models, enrichment of many KOG categories was observed (Fig. 3Sa), including defense mechanisms, secondary metabolites biosynthesis, and the category with no KOG classification or with function unknown. The enriched GO categories were involved in oxidoreductase activities and metal ion-binding (Table 4S-Sheet1).

Some (357) gene models found in the OmZn reference genome were not covered by reads of the 10 metal sensitive isolates, whereas they were covered by reads from the 5 Ni/Cr-tolerant isolates (Table 3S-Sheet 2). These gene models were enriched in KOG categories related to defense mechanisms and in the category with no KOG assigned (Fig.3Sb, Table 3S-Sheet2). The enriched GO-categories for these gene models were related to carboxylic ester hydrolase activity (Table 4S-Sheet2).

By contrast, 912 gene models from the OmZn reference genome were not covered by reads of the 5 Ni/Cr-tolerant isolates but were covered by the reads from the metal sensitive isolates. Enriched KOG categories were secondary metabolites biosynthesis and the category with no KOG (Fig. 3Sc, Table 3S-Sheet3). The enriched GO-categories of these gene models were the iron-binding, the heme-binding, the monooxygenase activity, and the oxidoreductase activity (Table 4S-Sheet3).

### Identification of single nucleotide polymorphisms (SNPs) in *O. maius* gene models

Comparisons with the reference OmZn genome highlighted a total of 1.8 × 10^6^ non-redundant SNPs across the 46.43 Mb genome, corresponding to 40,380 SNPs/Mbp and to 112.2 SNPs/gene (Table 2S). The SNPs were spread throughout the genome (Fig. 4S) and were equally distributed among coding and non-coding regions (Table 2S), ranging from 3 (in OmZn itself) to 22,068 SNPs/Mbp (in Om539) in the different isolates. The isolate with the lowest number of SNPs was OmCd (178 SNPs), while the isolate with the highest number of SNPs was Om539, with over 10^6^ SNPs. A Neighbor Joining tree computed by using all the SNPs showed that all tolerant isolates grouped in a single clade, separate from the sensitive ones (Fig. 2).

For 3,925 gene models, the absence of SNPs and/or lack of coverage did not allow calculation of the F_st_ value. The average F_st_ value calculated for the remaining 12,778 gene models was 0.44. Among them, 1,618 gene models showed a higher or equal F_st_ value when isolates were grouped according to their geographic origin rather than according to their metal-tolerant phenotype. These gene models were not considered further. Only 4,817 gene models met the following two criteria and were considered for subsequent analyses: F_st_ values based on metal tolerance groupings were larger than 0.5 and larger than F_st_ values based on geographic origin groupings (Fig. 5S, Table 5S-Sheet2). Among these gene models, the following KOG categories were significantly enriched: intracellular trafficking and secretion, protein modification and turn-over, nuclear processes such as cell cycle control, RNA processing and modification, chromatin structure, replication, recombination and repair, transcription and translation, transport activities involving nucleic acids, amino acids, lipids and carbohydrates and inorganic ions (Fig. 6S; Fisher test p-value ≤ 0.05). The GO-categories analysis showed that the following groups of functions/cellular components were over-represented in these gene models: DNA/RNA metabolism and modification, chromosome/chromatin organization, protein biosynthesis, metabolism and function, energy consumption/transfer, mitochondrion (Table 4S-Sheet4 for detailed GO terms). Among the 4,817 highly polymorphic gene models, 474 showed a dN/dS ratio above 1. Among these genes, only 274 were annotated, with the enrichment of two GO biological processes related to each other: nucleobase-containing compound metabolic process and nucleic acid metabolic process.

The Tajima’s D (D) value was successfully calculated for 12,890 gene models out of 16,703, with an average of 0.805. For 25 genes, the calculated D value was higher than 2, only enriched for the noKog-assigned genes category (p-val = 0.0146). For 62 genes, D value was lower than −2, with the enrichment of the noKog-assigned genes category (p-val = 0.0000569) and the KOG category related to cytoskeleton (p-val = 0.0094; Table 5S-Sheet3). No GO terms were enriched among these genes. One gene model (protein ID: 59318) showing a D value higher than 2, encoded for a trypsin-like peptidase and was found among the top-F_st_ genes as well.

### Identification of the most polymorphic genomic regions in metal tolerant and sensitive isolates

To analyze the genetic divergence of the full genome, including the non-coding regions, the F_st_ value was calculated for 3,305 10 Kb windows out of 4,693 over the entire genome, with an average value of 0.443 (Fig. 7S). When the isolates were grouped for their phenotype on metals, 1,845 windows showed a higher F_st_ value than when they were grouped for their geographical origin (Table 6S-Sheet1). Among these 1,845 windows, 745 had a F_st_ value higher than 0.5 (highly divergent) (Fig. 3, lower panel), and 60% of the genes included in such windows (1,881 out of 3,162 genes) had F_st_ values > 0.5 as well (Fig. 3, upper panel). Four genomic windows among the 1,845 mentioned above were statistical outliers, with F_st_ values > 0.726 (Fig. 3, pentagons in the lower panel; Table 6S-Sheet2). These 4 highly polymorphic windows harbored 19 gene models (Table 6S-Sheet3) and, among them, 10 genes were also among the 4,817 with F_st_ values > 0.5 (Fig. 3, pentagons in the upper panel), 5 with no GO function, the others belonging to the following GO categories: DNA binding, lactoylglutathione lyase activity, nitrilase activity, amino acid transmembrane transporter activity, carboxy-lyase activity.

The D value was calculated for 4,039 windows. Among them, one genomic window harboring three genes models had D value > 2 (balancing selection) (Table 6S-Sheet4), but only one gene model had D value > 2 and F_st_ value > 0.5, with no predicted function (Table 6S-Sheet5). A D value < −2 (positive selection) was found for 27 genomic windows (Table 6S-Sheet4) harboring 67 gene models, eight of which had D values < −2, with functions related to the cytoskeleton, RNA binding, electron transport, amino oxidase, and with unknown functions (Table 6S-Sheet5).

## Discussion

Chemically-stressed environments often host tolerant plant ecotypes capable of withstanding the toxic compounds (Muszyńska et al. 2019; Papadopulos et al. 2021). Among soil microbes, metal tolerant isolates of ectomycorrhizal fungi have been also identified in metal polluted soils (Branco et al. 2022; Jourand et al. 2010; Colpaert et al. 2004). Here, we confirmed this trend, showing that the metal tolerant/sensitive phenotype found in vitro for the studied *O. maius* isolates mirrored in most cases their provenance from metal-polluted and unpolluted soils, with isolates from polluted soils showing a higher heavy metal tolerance than those from unpolluted or mildly polluted soils, thus suggesting that a selection process possibly occurred in contaminated soils. Interestingly, the top Ni-tolerant isolate grew better on Ni-amended medium than in control conditions. This result emphasises the fact that some isolates have not only adapted successfully to the site contaminants, but that these elements are important to support their growth. Nickel, although it can become toxic above a given threshold concentration, is an essential micronutrient for microorganisms and plants, where different nickel-containing enzymes were identified that participate in several cellular processes (Mulrooney & Hausinger 2003).

The genetic divergence between metal tolerant and metal sensitive *O. maius* isolates was high and relied on polymorphisms that concern about one third of the gene models in the genome and non-coding regions.

### The SNPs-based phylogeny clustering mirrors the isolates’ metal tolerance

*O. maius* isolates exhibited a genetic diversity higher than other fungi from previous papers, and our analyses showed that the isolates grouped according to their ability to tolerate heavy metals in the medium. Indeed, a phylogeny built using all SNPs showed that the tolerant isolates clustered in two groups corresponding to the metals they were tolerant to, i.e. Zn / Cd or Ni / Cr. This suggests that experiments in vitro may elucidate the role of SNPs in the adaptation of each isolate to the environment (Stukenbrock 2013). Besides, the phylogenetic analysis based on the ITS2 sequences of each isolate supported the morphological identification of the 18 *O. maius* isolates and suggested that the taxonomic distance among the isolates does not mirror their metal tolerant phenotype, nor their geographical origin. The *O. maius* isolates exhibited a high level of genetic diversity, with a total of 40,380 SNPs/Mbp (total number of non redundant SNPs / length of the genome). This density of SNPs is similar (43,126 SNPs/Mbp) to that of *S. luteus* isolates from polluted and unpolluted sites (Bazzicalupo et al. 2020). The density of SNPs found in isolates of other fungal species from geographically distant sites and/or subject to different environmental conditions was in general lower: 3,540 SNPs/Mbp for *Tuber melanosporum* (Payen et al. 2015), 28,377 SNPs/Mbp for *Suillus brevipes* (Branco et al. 2017), 10,243 SNPs/Mbp for *Melampsora larici-populina* (Persoons et al. 2022). The presence of heavy metals in the soil could therefore increase the mutation rate and possibly shift the balance between mutation rate and selective pressure against weakly purifying selection. Indeed, it has been proposed that a fungal species exposed to stress can evolve quickly, such as *Gastrosuillus larcinus* that differentiated from *Suillus grevillei* in a few decades (Baura et al 1992).

The experimental plots in the Niepolomice Forest, from which the *O. maius* tolerant isolates have been isolated, have been subjected to an intense artificial contamination of heavy metal containing dusts (from 100 up to 5000 tons/km^2^ x year, distributed in 4 doses in 1980). These fungi have been isolated six to nine years later (Turnau 1991), after the recolonization of the contaminated plots by *V. myrtillus*, one of the first species appearing after the treatment. This species belongs to the ericaceous family and is known to colonize low-pH, heavy metal rich soils, with ericoid mycorrhizal fungi playing a key role by enhancing the plant tolerance to heavy metal stress (Casarrubia et al. 2020; Perotto et al. 2012). Some of these fungal isolates were already identified as being metal tolerant in previous work (Martino et al. 2003), with OmZn and OmCd being the top growing strains in the presence of Zn, like in our study.

No data are available on the *O. maius* population inhabiting that soil before the contamination, but one metal-sensitive isolate (*O. maius* A) was isolated from a non-contaminated soil plot in the same site. Therefore, it is tempting to speculate that *O. maius* isolates have rapidly adapted, by mechanisms including nucleotide substitutions, to tolerate this intense contamination, possibly allowing the colonization of the contaminated soils by their  host plant. Several years of subculture using media without metal did not change the tolerant phenotype of these *O. maius* isolates (e.g. Martino et al. 2000a, 2003; Vallino et al. 2011), further suggesting a stable genetic modification leading to metal tolerance.

OmCd showed a very low number of SNPs when mapped on the reference genome, thus suggesting that it may be a clone of the reference isolate OmZn, although the phenotypes recorded here and in previous publications were similar but not identical (Martino et al. 2003; Vallino et al. 2011). They also host the same mycovirus (Sutela et al. 2020) and this observation raises, for future research, intriguing questions about the possible role of mycoviruses in the metal tolerant phenotype of both ErM fungi and their host plants, since mycoviruses in endophytic fungi are known to improve stress tolerance of their fungal host, but also of the colonized host plant (Marquez et al. 2007).

### The genome of the Zn-tolerant isolate OmZn harbors gene models that were not detected in the other fungal genomes

Our identification of gene models of the reference strain that are not covered by reads from the other strains, with some differences between sensitive and tolerant ones, suggests the importance of gene duplication/deletion in the evolution of these different phenotypes. The regions of the reference OmZn genome that were not covered by any reads from the other *O. maius* isolates may either correspond to missing regions in the re-sequenced genomes or to highly polymorphic regions that fall out of the parameters used for reads’ mapping (Payen et al. 2015). Although a de novo sequencing of these new isolates would be necessary in order to definitely clarify this point, the lack of reads mapping across such regions in all the isolates except OmCd and Om505, the two other Zn/Cd tolerant isolates, may suggest that the data is not merely due to a poor assembly/mapping. Indeed, all the isolates had some missing/highly polymorphic regions, within which we found a number of coding sequences. Among the 930 gene models missing in all isolates (except OmZn, OmCd and Om505), 11 were transcriptionally regulated in the OmZn mycelium exposed to CdSO_4_ (Casarrubia et al. 2020), 6 with functions involved in NADH metabolism and energy production, cytoskeleton and inorganic ion transport (in red in Table S3 Sheet1, data from Casarrubia et al. 2020), including a gene predicted as FAD-binding ferredoxin-reductase-type domain-containing protein, that putatively oxidizes ferredoxin to produce NADH. Interestingly, the UNIPROT blast search of this protein revealed the presence, in the genome of OmZn, of a close relative with 87% of sequence identity, suggesting that they may be paralogs derived from a duplication event. Further analyses are needed to test this hypothesis, but gene duplication is a major force driving adaptive innovation in fungi and the copy-number variation in stress-responsive genes may be beneficial, allowing adaptation to diverse ecological niches (Gladieux et al. 2014; Wapinski et al. 2007). For example, heavy metal tolerance in fungi is often achieved through gene copy number variation, as demonstrated in *S. luteus* (Bazzicalupo et al. 2020). An inter-species comparative genomics study on *Aspergillus* spp. demonstrated that different levels of stress tolerance were often associated with the presence of specific genes that were absent in the sensitive species, such as a new type of *SodA* in the oxidative-stress tolerant *A. brasiliensis*, or the Pca1-type Cd(II) transporter in the genomes of the most Cd(II)-tolerant species (de Vries et al. 2017). Gene deletion and horizontal gene transfer are important mechanisms of adaptation as well (Gladieux et al. 2014; Novo et al. 2009; Cheeseman et al. 2014), that might explain the lack of hundreds of genes in the *O. maius* isolates different from the reference.

Genes with no predicted function were over-represented across the non-mapped regions and some of them were found to be up-regulated in OmZn exposed to Cd (Casarrubia et al. 2020), suggesting a role in metal tolerance. In addition, 122 genes missing in all the new isolates may be defined as “orphans” since they were not present in the genomes of 60 other fungi from different lineages (Martino et al. 2018). This suggests that the genetic difference between OmZn and the other *O. maius* isolates could also involve many unknown functions that may be crucial for its phenotype.

Genes belonging to the “defense mechanisms” KOG class were over-represented among those specifically missing in all the metal sensitive isolates, and four gene models among them were transcriptionally regulated by CdSO_4_ (in red in Table S3 Sheet2; Casarrubia et al. 2020). The same KOG class was not over-represented among the genes specifically missing in all the metal tolerant isolates. This observation suggests a higher readiness of the metal tolerant isolates with the heavy metal tolerant reference, as far as concerns genes involved in defense mechanisms.

### A high genetic divergence between metal tolerant and metal sensitive isolates is detected in one third of the gene models

The indicators of genetic divergence and neutrality in evolution suggest that the divergence between tolerant and sensitive isolates resides in a wide portion of the genome, including both gene models and intergenic/non-coding regions.

The F_st_ value was calculated to identify genes and genomic regions under genetic differentiation (Stukenbrock 2013). The F_st_ value calculated for each gene model was on average high, indicating a high genetic differentiation between the two groups of isolates (tolerant *vs* sensitive) concerning genes throughout the whole genome. The identification of genes with the highest genetic differentiation was done by considering the accepted threshold in the literature (F_st_ > 0.5, Hartl & Clark 1997). Around one third of the *O. maius* genes displayed a high genetic differentiation, differently from what was observed when comparing geographically separated fungal populations (Branco et al. 2015) or geographically close populations from polluted and unpolluted soils (Bazzicalupo et al. 2020), whose genomes showed few localized highly diverging regions.

In addition, the divergence estimates F_st_ calculated on the complete *O. maius* genome showed a genome-wide distribution consistent with the distribution computed on the gene models only. The majority (60%) of the gene models harbored in the most diverging 10 Kb regions had F_st_ > 0.5, thus being very polymorphic as well. On the other hand, we found conserved non-polymorphic gene models within polymorphic non-coding regions that might play a role by controlling gene expression in response to metal stress. This was also observed in a previous paper for the *Sod1* locus (Vallino et al. 2011) and could be confirmed here for the same locus in those isolates that were considered in both works, e.g. from the Poland contaminated Niepolomice forest and from unpolluted sites. Indeed, previous papers report that promoters for essential and housekeeping genes are more stable to mutation, while promoters for genes whose expression is regulated by external stimuli, like stress-response and effector genes, are more labile and subject to rapid evolution (Tirosh et al. 2006; Gladieux et al. 2014), playing a possible role in the development of a tolerant phenotype.

A D value below − 2 or above 2 is generally considered to be a strong indication that a gene is not evolving neutrally. Only 25 genes and 1 genomic window showed a D statistic index over 2, suggestive of balancing selection retaining genetic diversity. This window included three gene models, but only one showed a high D value, likely being affected by the selection. 62 gene models showed a D statistic lower than −2, as well as 27 genomic windows, indicating positive selective pressure, where an advantageous variant has recently replaced most of the variation in a region (selective sweep). These genome regions harbored either gene models under selection or neutral ones, the latter data suggesting that the noncoding sequences also might be under selection.

### Gene loss or high polymorphism: predicted functions revealed by genome scan of tolerant and sensitive isolates

The genome scan of *O. maius* isolates showing different heavy metal tolerance allowed the identification of mutations and possible gene loss that may underlie a functional adaptation with no need of a priori definition. We calculated one divergence statistic (F_st_) and one index measuring bias in SNP frequencies (D) and, given the high number of involved gene models and genome windows, we crossed the lists in order to take out the most significant data. Among those genes, some of the most polymorphic ones showed homology with genes with known functions in other organisms, and often the predicted functions suggest a possible role in heavy metal tolerance. In particular, functions related to amino acid metabolism, protein turnover and transcription regulation by epigenetic mechanisms (e.g. chromatin remodeling, ncRNA processing) appear to be very abundant. In addition, some enzymes with hydrolytic functions are among the most polymorphic or the missing ones, and may have a role in the interaction of *O. maius* tolerant strains with the substrate, supporting the strong saprotrophic ability of *O. maius* and providing an indirect advantage in unfavorable growth conditions, like in the presence of heavy metals (Martino et al. 2000b). The genes, cellular functions/components affected by one of the described genetic changes are discussed below.

*Transcription and epigenetic control of expression*: Among the genes with high F_st_ values, the enrichment of GO terms related to RNA, DNA and chromatin reorganization, including ncRNA processing, macromolecules methylation and histone modification, suggests that the selective pressure over epigenetic mechanisms might contribute to the heavy metal tolerant phenotype development. Heavy metal tolerance mechanisms controlled by epigenetic processes have been already reported for plants (Gullì et al. 2018; Niedziela 2018), earthworms (Kille et al. 2013) and animals, including humans (Mani et al. 2019).

*Mitochondrion*: The enrichment of mitochondrion related genes among the divergent gene models and the Cd-upregulated divergent genes (Casarrubia et al. 2020) is in line with previous results suggesting the strong involvement of the mitochondrion in the OmZn heavy metal tolerance mechanisms (Daghino et al. 2019). Indeed, Cd toxicity was reported to disrupt the mitochondrial membrane potential, also inducing ROS formation, in *Phanerochaete chrysosporium* (Chen et al. 2014).

*Nitrogen metabolism:* A flavin containing amino oxidase displayed a low D and was included in a low D region. In fungi these enzymes provide a source of ammonium (Schilling and Lerch 1995) while in plants they are involved in the catabolism of polyamines (Tavladoraki et al. 1998). In addition, a *NmrA*-like gene was found among the non-mapped genes, therefore likely absent in the sensitive isolates. As NmrA is a negative transcriptional regulator involved in the control of the nitrogen metabolism in fungi (Han et al. 2016), this finding supports the importance of this primary metabolism in heavy metal tolerance, as already suggested by the deletion of the glutamine oxoglutarate aminotransferase (*GOGAT)* gene from OmZn, leading to the loss of metal tolerance (Khouja et al. 2014).

*Enzymes with hydrolytic activities*: Four α/β-hydrolases coding genes were absent/highly polymorphic in the sensitive isolates’ genomes. These enzymes may play a role in soil organic matter degradation (Jochens et al. 2011), thus providing the tolerant isolates with a nutritional advantage that could also translate to improved growth in heavy metals contaminated environments. Indeed, a previous paper demonstrated that the activity of polygalacturonases, hydrolytic pectin degrading enzymes, was induced by Zn and Cd in the OmZn and OmCd isolates (Martino et al. 2000b). A trypsin-like serine and cysteine peptidase with high F_st_ and D values was detected, indicating a high divergence between the two groups in this locus and a diversifying selection (Tajima 1989). In bean cotyledons, copper exposure was reported to increase trypsin-like activity suggesting a protective role against copper stress played by these endoproteases (Karmous et al. 2014). In addition, the Cys-peptidases include phytochelatin synthases, that are involved in the synthesis of heavy metal chelating molecules in plants and fungi (Hasan et al. 2017; Shine et al. 2015).

*Amino acid transport and metabolism, secondary metabolites biosynthesis:* Four highly diverging genes, hosted in highly polymorphic genomic surroundings, were involved in amino acid transport and metabolism: a nitrilase/cyanide hydratase function containing enzyme, a carboxy-lyase function containing enzyme, a D-3-phosphoglycerate dehydrogenase and an amino acid permease. Interestingly, the D-3-phosphoglycerate dehydrogenase is involved in the synthesis pathway of L-serine, a precursor of both cysteine and glycine, needed for the synthesis of glutathione and phytochelatins, important metabolites for heavy metal tolerance in plants and fungi (Ruytinx et al. 2016). L-serine is also the precursor for choline and glycine betaine biosynthesis in plants and fungi (Hai et al. 2019). Glycine betaine plays an essential role in abiotic stress protection, either by the osmotic control of water absorption or by the reactive oxygen species (ROS) scavenging (Lambou et al. 2013), ROS detoxification being among the known defenses from heavy metal damages. The main precursor for glycine betaine biosynthesis is choline. Interestingly the amino acid permease, is predicted to belong to the amino acid/polyamine transporter family and shares sequence similarities with the choline-transporters from other Helotiales (i.e. *Cadophora* sp., *Lachnellula occidentalis*; Knapp et al. 2018). Concerning polyamines, their accumulation in response to Pb and Zn was observed in the ectomycorrhizal fungus *Paxillus involutus* (Zarb and Walters 1996). In OmZn, the upregulation of agmatinase, an enzyme involved in polyamine biosynthesis, was observed upon exposure to Zn and Cd (Chiapello et al. 2015). Furthermore, one of the missing genes in the sensitive *O. maius* isolates, and up-regulated by Cd in OmZn, was predicted to be an ornithine carbamoyltransferase, a mitochondrial enzyme synthesizing citrulline, the precursor of arginine and polyamines (Dzikowska et al. 2015).

*Stress tolerance and antioxidant defenses:* antioxidant defenses are crucial in fungi heavy metal tolerance (Ruitinx et al. 2016). Functions related to antioxidant defense, including oxidoreductase activity and alcohol dehydrogenase, were found among the most polymorphic genes. Overall, the “defense mechanism” KOG class was enriched among genes missing in all sensitive isolates, likely being involved in the adaptation of tolerant strains, through gene duplication and polymorphism. One of the highly polymorphic windows harbored a polymorphic glyoxalase gene, coding for an enzyme that catalyzes the detoxification of methylglyoxal, known to play a crucial role in plant heavy metal stress tolerance and pathogen resistance (Yan et al. 2018).

*Intracellular trafficking, transmembrane transporters and secretion:* the KOG class grouping functions related to intracellular trafficking, secretion and vesicular transport was enriched among the most polymorphic genes, as well as the GO term “vesicle-mediated transport”. Uptake/efflux, transport and compartmentation mechanisms play a primary role in fungi for maintaining metal homeostasis. The transmembrane fluxes through endoplasmic reticulum localized transporters might be involved in the removal of excess cytoplasmic Zn in the fungus *Hebeloma cylindrosporum* (Blaudez and Chalot 2011). Also, two metal-transporters from OmZn rescued the metal tolerant phenotype in mutant yeast strains (Khouja et al. 2013), with the gene coding for one of them, *OmZnT1*, diverging compared to those of sensitive isolates having an F_st_ value > 0.5. Most probably only a few transporter-coding genes play crucial roles in the tolerant phenotype, since the “inorganic ion transport” KOG class was not enriched in any of our analyses.

## Conclusions

The overall picture from this genome scan shows a number of genes strongly diverging between the groups of metal tolerant and metal sensitive *O. maius* isolates. Many genes in the metal tolerant reference OmZn were missing or were highly divergent in the other isolates, suggesting that the tolerant phenotype was not derived by a single adaptation mechanism, but phenotypic plasticity (i.e. the ability of a genotype to produce different phenotypes under different environmental conditions thanks to a differential gene expression, explained in some cases by epigenetics (Kronholm et al. 2016)) cannot be excluded. Another interesting point to be considered is that heavy metals can induce genome instability. In humans, some heavy metals such as Cd, are known to induce mutagenicity and genetic instability bringing to ROS formation and inhibiting DNA repair mechanisms (Filipič et al. 2006). The genome of fungi exposed to heavy metal has also been reported to exhibit high genome instability through mutation accumulation (Collin-Hansen et al. 2005), possibly leading to higher genetic diversity for strains coming from contaminated sites. It has also been shown that in case of gene flow between populations from contaminated soils versus those of non contaminated soils, genetic diversity could be similar, but genetic adaptation could select some specific genomic regions (Bazzicalupo et al. 2020). Heavy metals could also induce genome evolution by increasing selection pressure (Bazzicalupo et al 2020).

According to our results we could conclude that, at least in part, the different phenotypes observed among the *O. maius* strains tested are due to genetic divergence, although additional epigenetic studies could be performed in future to investigate phenotypic plasticity either, also given the strong representation of related functions among the most diverging genes.

Similar to *O. maius*, heavy metal tolerance of *S. luteus* was also found to be a polygenic trait (Bazzicalupo et al. 2020), although with a lower average genetic divergence. The high level of genomic divergence detected between sensitive and tolerant strains likely suggests a faster evolution for the latter strains, that might be a consequence of the exposure to Zn, an oxidative metal, and Cd, that can cause strong damages to membranes, proteins and nucleic acids (Robinson et al. 2021; Genchi et al. 2020). Also, many functions are involved in the adaptation to heavy metals and these functions vary for different metals (Colpaert 2008). Indeed, the overall genetic difference between the isolates, both for SNPs distribution and the absence of many gene models in their genome, mirrored the ability of the analysed isolates to grow in the presence of different metals.

Functional studies are required to elucidate the exact role of genes located in polymorphic regions, as well as genes found only in the tolerant isolates. Some highly diverging genes might be promising candidates for mutation experiments, such as the above-mentioned trypsin-like serine and cysteine peptidase.

The de novo assembly and annotation of sensitive isolates will help in the understanding of the genetic differences between the two groups, including possible gene gain/loss events. Many polymorphic genes have no known function, but the increase of available fungal genomes will help in the future to elucidate their function. Also, the high polymorphism of genes coding for hydrolytic enzymes in tolerant strains is intriguing. Indeed in the harsh environments hosting ErM plants, with highly complex organic substrates possibly contaminated by heavy metals, ericoid fungal ability to both tolerate these toxic compounds while also degrading complex organic matter is compulsory for plants’ survival, providing them with the ability to dominate these hostile habitats. The results of this and future genomic analyses on heavy metal tolerant and sensitive ErM fungi, that might have different protective effects on their host plant, might be also helpful in clarifying the involved molecular functions.

## Supplementary Information

Below is the link to the electronic supplementary material.

Supplementary file1 Phylogenetic analysis of ITS sequences in the Oidiodendron genus, showing that all the strains considered in this study belong to the O. maius species. The metal-sensitive isolates are in green, while the tolerants are in red. The globe color indicates the geographic origin as described by the legend (PNG 201 KB)

ESM 2 Biomass of the isolates grown on heavy metal amended media. The result is expressed as ratio of the biomass obtained in the presence of heavy metals (T) and in the absence of them (C ). The bars span from the maximum to the minimum value, the square is the average and the horizontal line within the boxes represents the 50^th^ percentile. Zn: ZnSO_4_٠7H_2_O 15 mM; Cd: 3CdSO_4_٠8H_2_O 0.3 mM; Cr: K_2_Cr2_2_O_7_ 0.6 mM; Ni: NiSO_4_٠7H_2_O 0.6 mM (PNG 14.4 KB)

Supplementary file2 (TIFF 3059 KB)

ESM 3 KOG classes of genes enriched among those that are not covered by any reads either (a) from all the other strains except OmZn, OmCd and OmCd505, or (b) from all the sensitive strains or (c) from the tolerant strains except OmZn, OmCd and OmCd505. In (a) only the enriched (p-val<0.05) are shown, while in (b) and (c) all the KOG categories found are reported and the enriched ones are indicated in the text (PNG 684 KB)

Supplementary file3 (TIFF 3660 KB)

ESM 4 Distribution of the SNPs in the genome. Each point represents the number of SNPs in a genomic window of 10Kb. Different colors indicate the position of the genomic windows in a different genome scaffold (PNG 3.59 MB)

Supplementary file4 (TIFF 2465 KB)

ESM 5 Distribution of the polymorphism differentiating sensitive and tolerant isolates. Each point represents the F_st_ value calculated per each gene model in the genome. Different colors indicate the position of the gene models in a different genome scaffold (PNG 2.09 MB)

Supplementary file5 (TIFF 1191 KB)

ESM 6 KOG classes that are enriched among the most polymorphic genes (4817 genes with F_st_>0.5, see text) with respect to the other genes (PNG 1.36 MB)

Supplementary file6 (TIFF 1322 KB)

ESM 7 Distribution of the polymorphism differentiating sensitive and tolerant isolates. Each point represents the Fst_st_ value calculated per each 10Kb genome window in the genome. Different colors indicate the position of the window in a different genome scaffold (PNG 1.64 MB)

Supplementary file7 (TIFF 1469 KB)

Supplementary file8 (XLSX 13 KB)

Supplementary file9 (XLSX 14 KB)

Supplementary file10 (XLSX 169 KB)

Supplementary file11 (XLSX 328 KB)

Supplementary file12 (XLSX 3612 KB)

Supplementary file13 (XLSX 281 KB)

## Data Availability

Sequence data that support the findings of this study have been deposited in the NCBI SRA sequence reads archive under Accession No PRJNA1101509.
