# Supplementary figures and images for: Comparative genomics reveals substantial divergence in metal sensitive and metal tolerant isolates of the ericoid mycorrhizal fungus Oidiodendron maius
Source: Mycorrhiza. 2025 Mar 21;35(2):24. doi: 10.1007/s00572-025-01191-x (PMC11928401; doi:10.1007/s00572-025-01191-x)

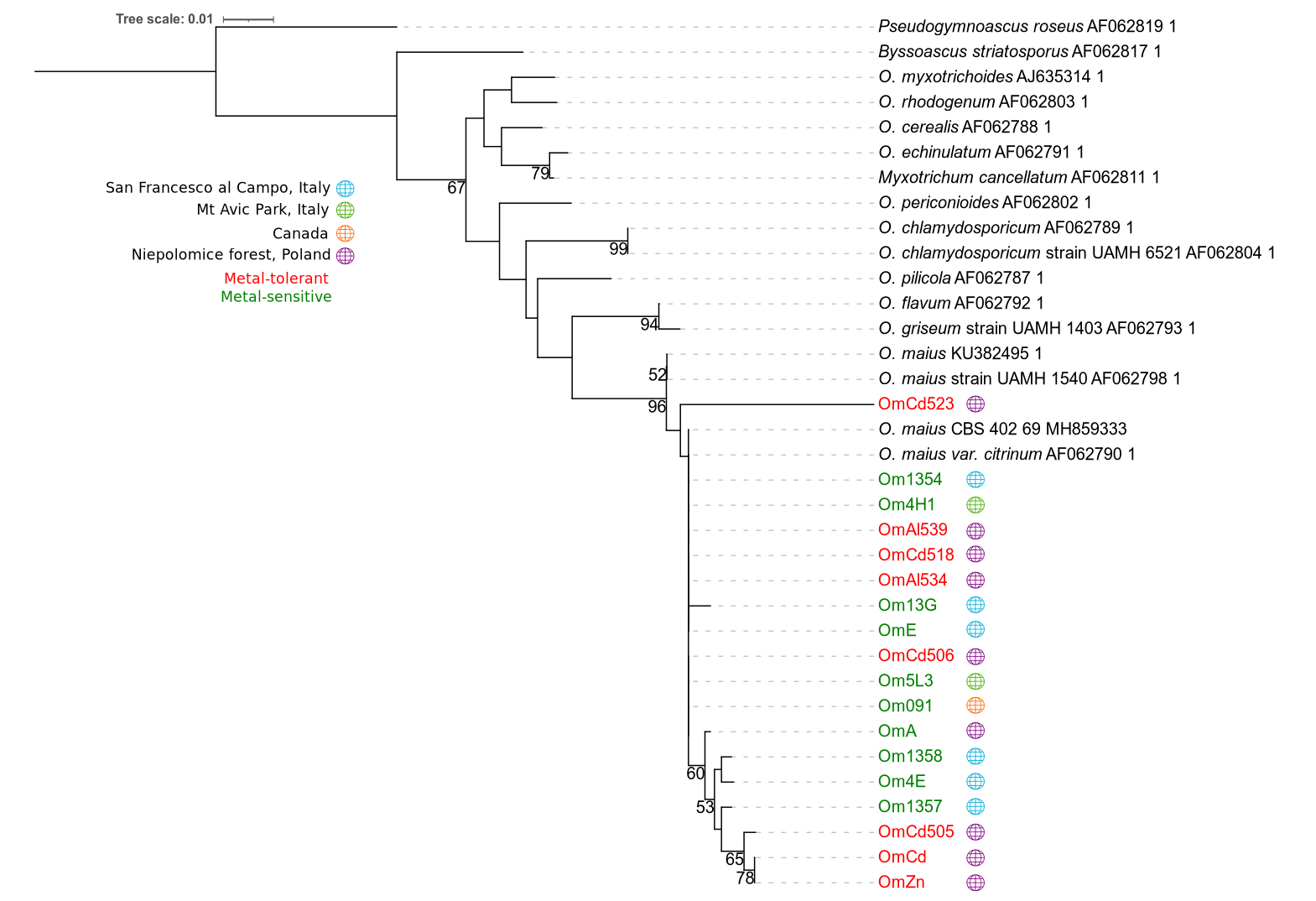

Supplement: Supplementary file 1 — Supplementary file1 Phylogenetic analysis of ITS sequences in the Oidiodendron genus, showing that all the strains considered in this study belong to the O. maius species. The metal-sensitive isolates are in green, while the tolerants are in red. The globe color indicates the geographic origin as described by the legend (PNG 201 KB) [file 572_2025_1191_MOESM1_ESM.png]

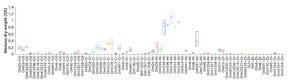

Supplement: Supplementary file 2 — Biomass of the isolates grown on heavy metal amended media. The result is expressed as ratio of the biomass obtained in the presence of heavy metals (T) and in the absence of them (C ). The bars span from the maximum to the minimum value, the square is the average and the horizontal line within the boxes represents the 50th percentile. Zn: ZnSO4٠7H2O 15 mM; Cd: 3CdSO4٠8H2O 0.3 mM; Cr: K2Cr22O7 0.6 mM; Ni: NiSO4٠7H2O 0.6 mM (PNG 14.4 KB) [file 572_2025_1191_Fig4_ESM.png]

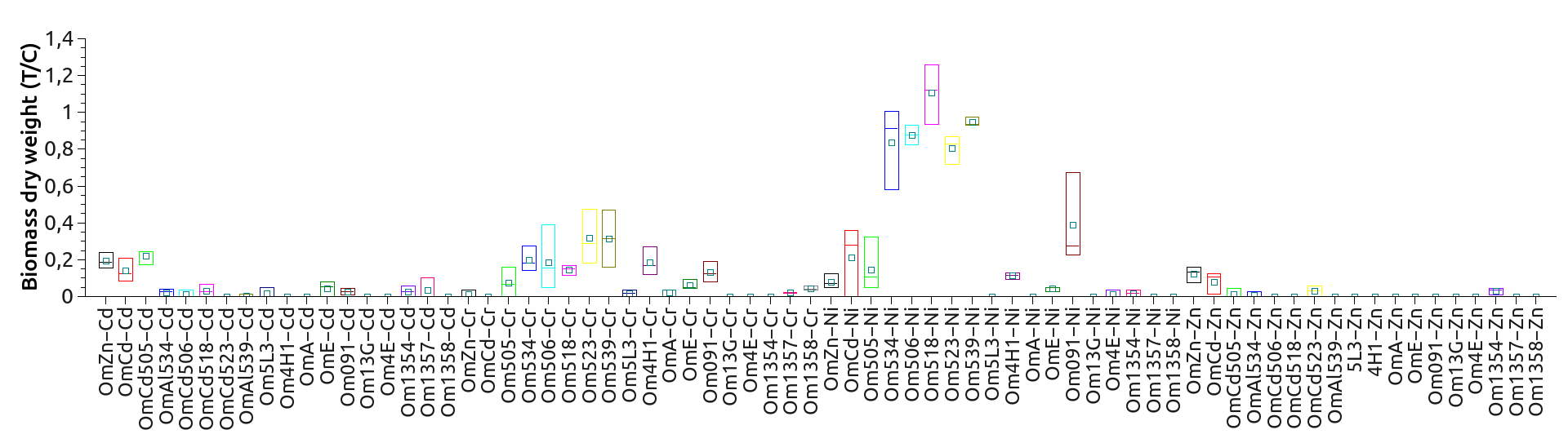

Supplement: Supplementary file 3 — Supplementary file2 (TIFF 3059 KB) [file 572_2025_1191_MOESM2_ESM.tiff]

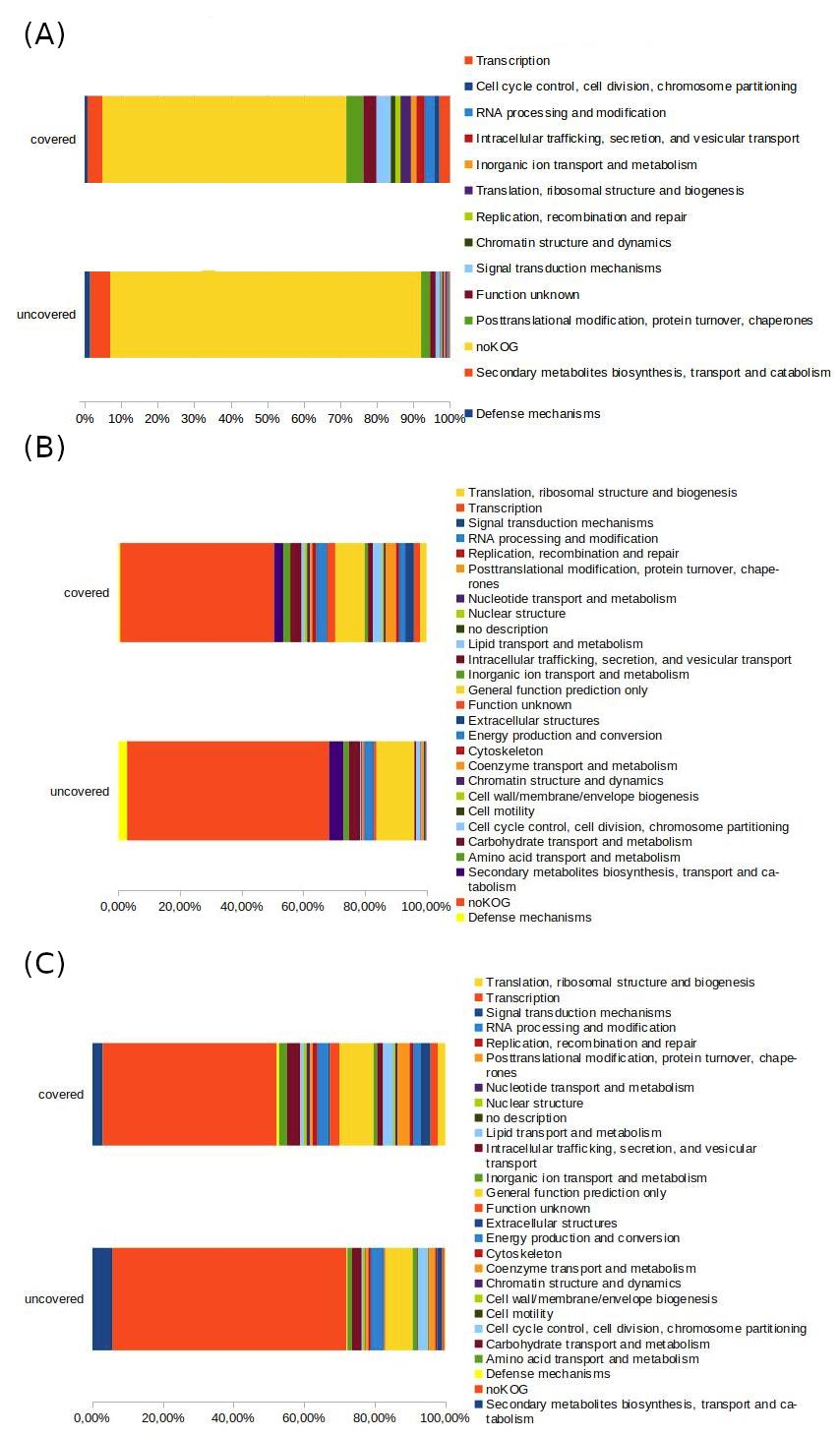

Supplement: Supplementary file 4 — KOG classes of genes enriched among those that are not covered by any reads either (a) from all the other strains except OmZn, OmCd and OmCd505, or (b) from all the sensitive strains or (c) from the tolerant strains except OmZn, OmCd and OmCd505. In (a) only the enriched (p-val<0.05) are shown, while in (b) and (c) all the KOG categories found are reported and the enriched ones are indicated in the text (PNG 684 KB) [file 572_2025_1191_Fig5_ESM.png]

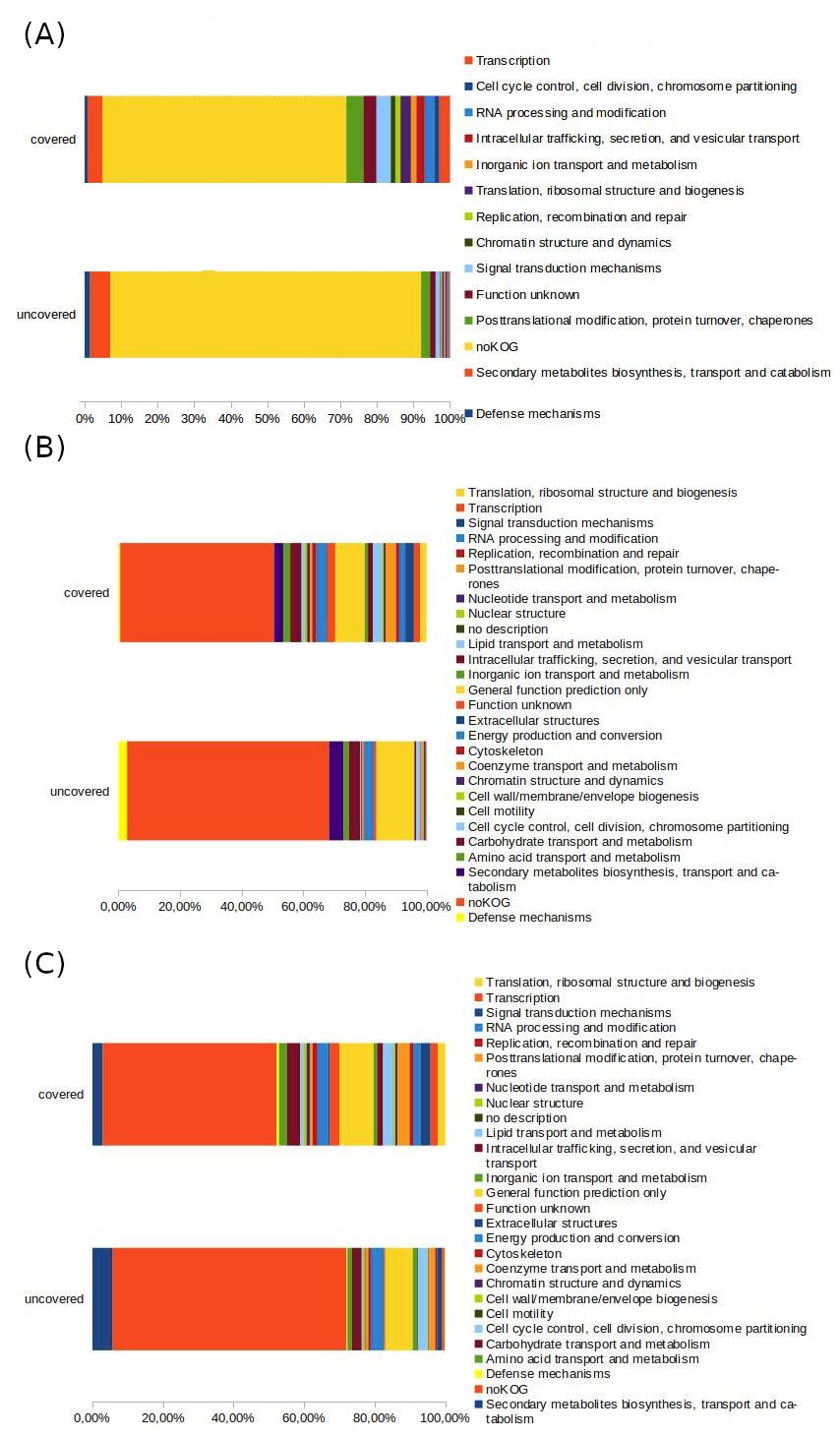

Supplement: Supplementary file 5 — Supplementary file3 (TIFF 3660 KB) [file 572_2025_1191_MOESM3_ESM.tiff]

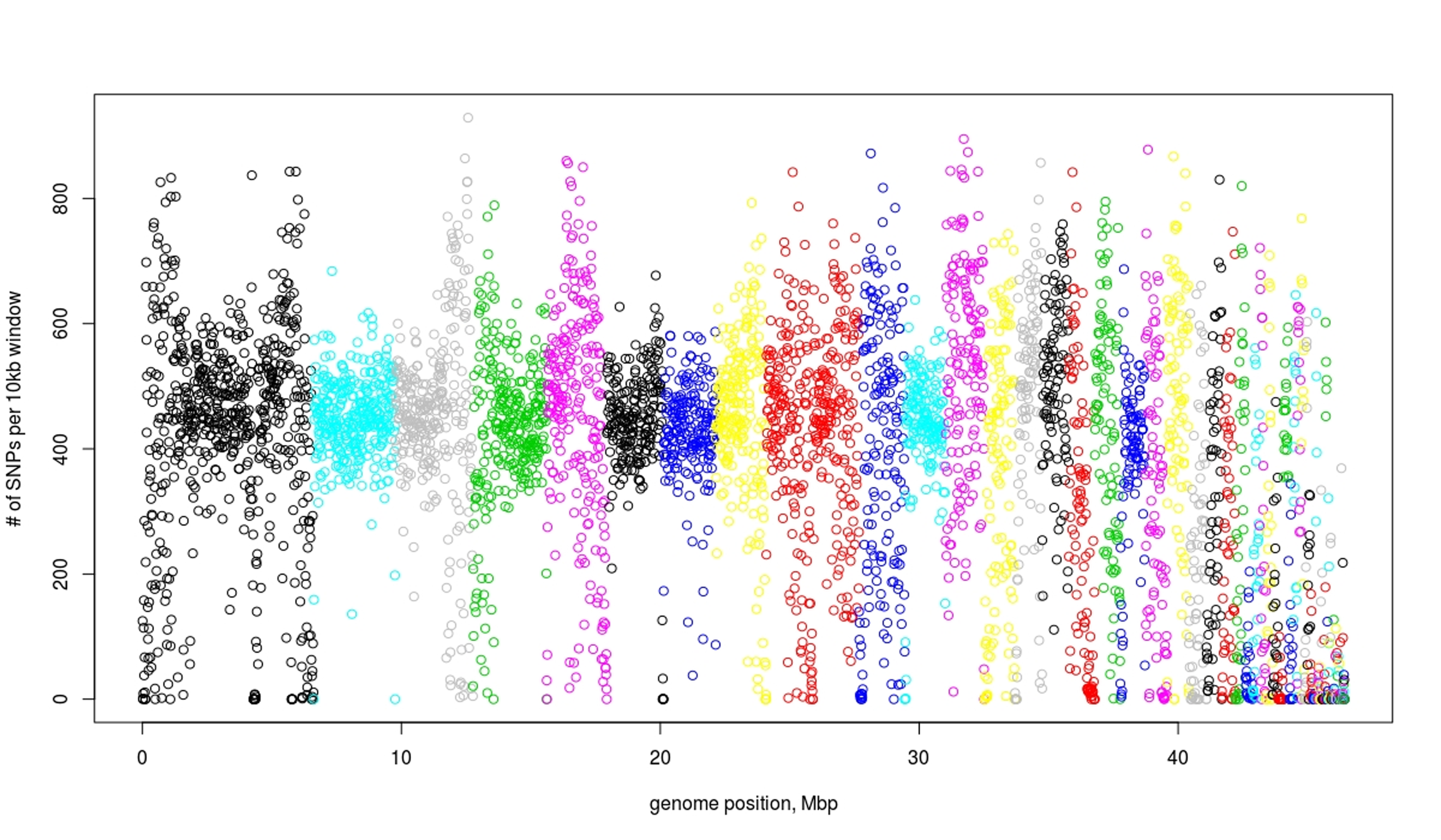

Supplement: Supplementary file 6 — Distribution of the SNPs in the genome. Each point represents the number of SNPs in a genomic window of 10Kb. Different colors indicate the position of the genomic windows in a different genome scaffold (PNG 3.59 MB) [file 572_2025_1191_Fig6_ESM.png]

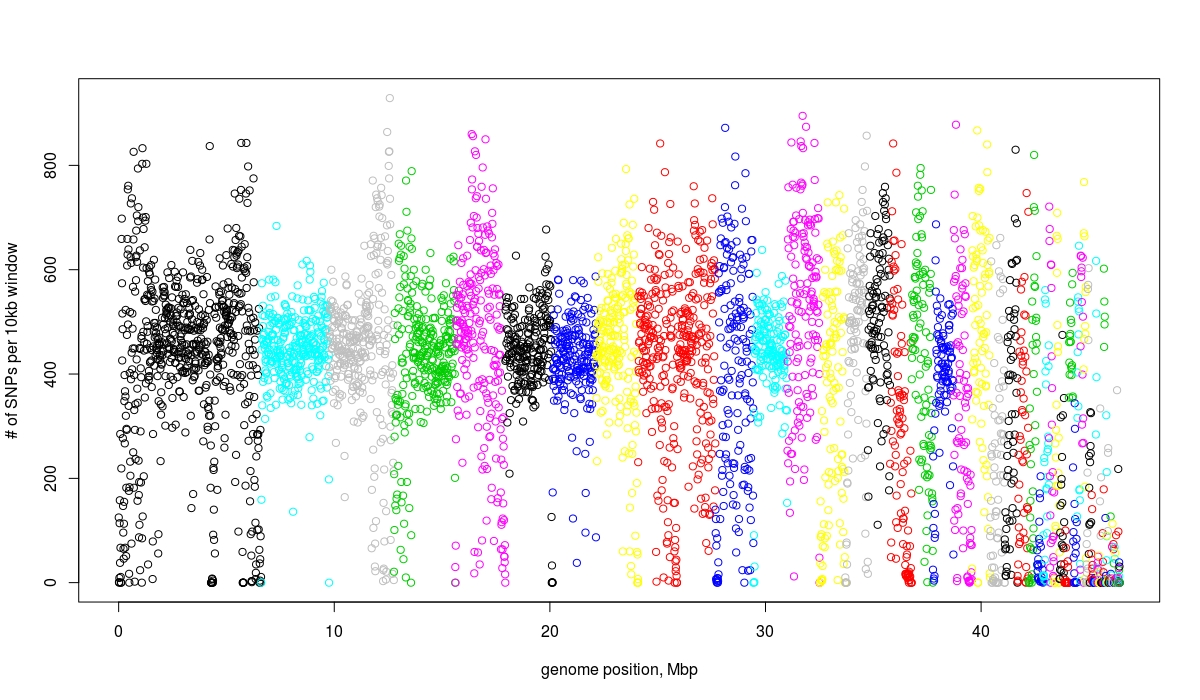

Supplement: Supplementary file 7 — Supplementary file4 (TIFF 2465 KB) [file 572_2025_1191_MOESM4_ESM.tiff]

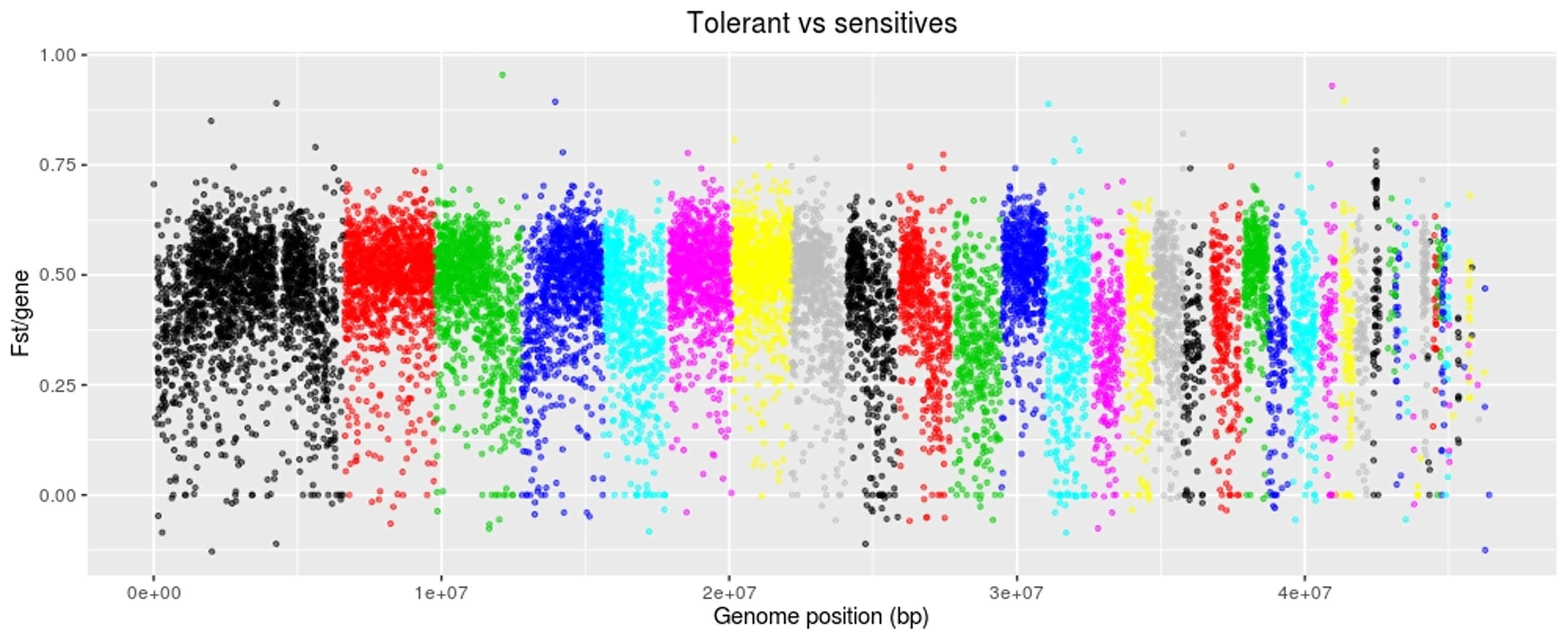

Supplement: Supplementary file 8 — Distribution of the polymorphism differentiating sensitive and tolerant isolates. Each point represents the Fst value calculated per each gene model in the genome. Different colors indicate the position of the gene models in a different genome scaffold (PNG 2.09 MB) [file 572_2025_1191_Fig7_ESM.png]

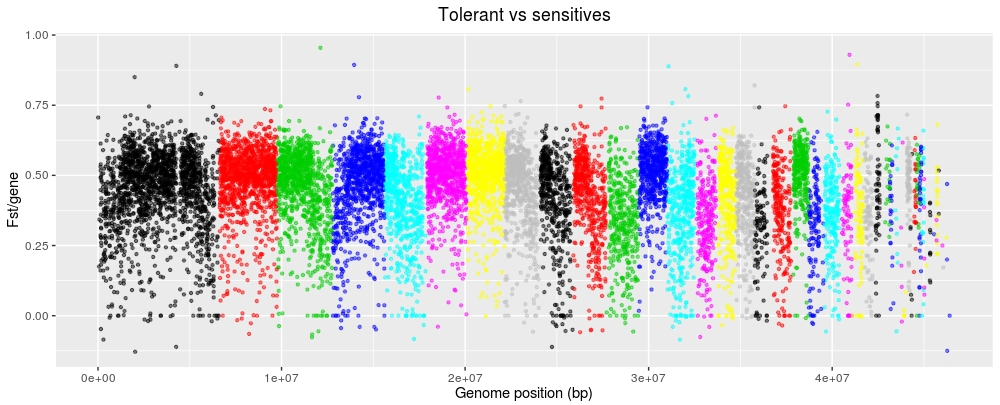

Supplement: Supplementary file 9 — Supplementary file5 (TIFF 1191 KB) [file 572_2025_1191_MOESM5_ESM.tiff]

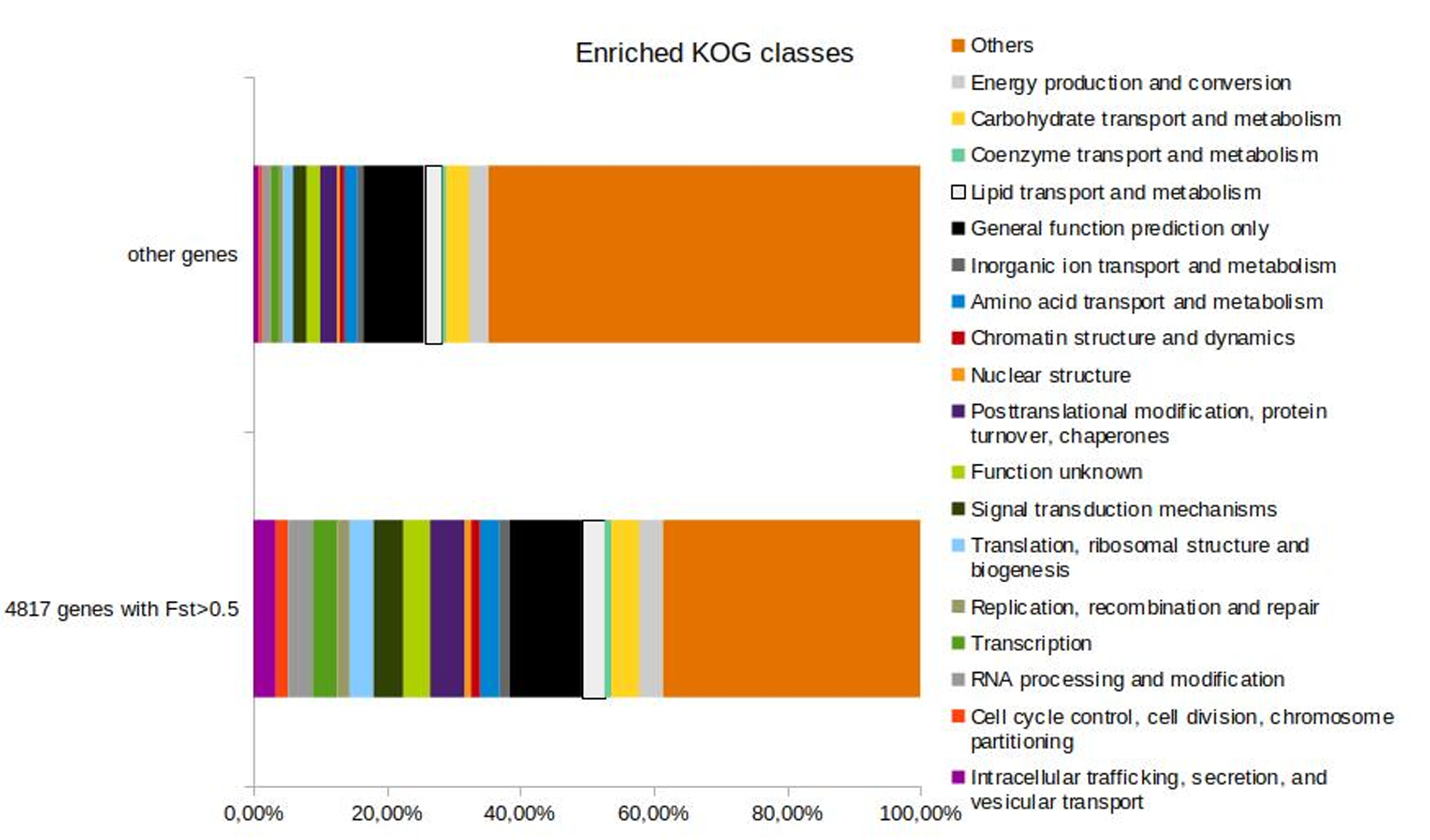

Supplement: Supplementary file 10 — KOG classes that are enriched among the most polymorphic genes (4817 genes with Fst>0.5, see text) with respect to the other genes (PNG 1.36 MB) [file 572_2025_1191_Fig8_ESM.png]

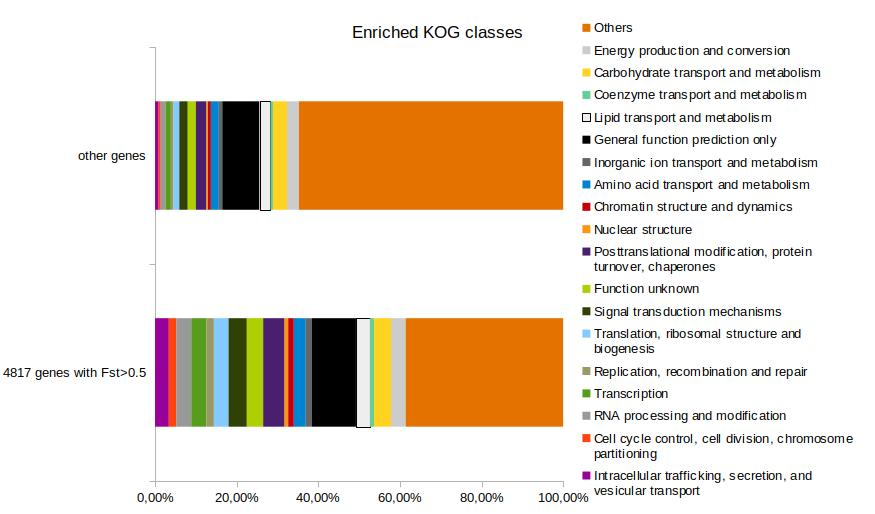

Supplement: Supplementary file 11 — Supplementary file6 (TIFF 1322 KB) [file 572_2025_1191_MOESM6_ESM.tiff]

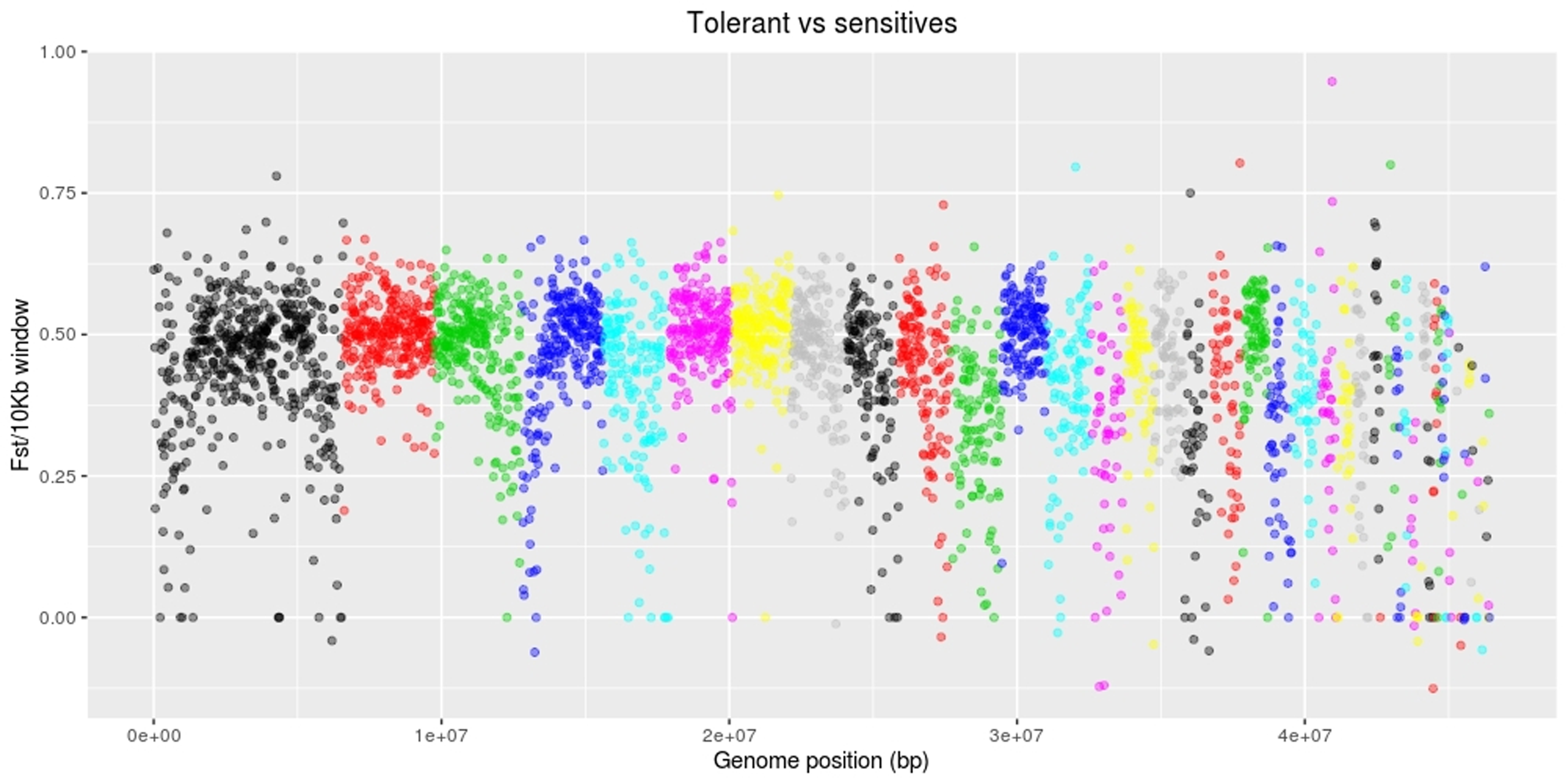

Supplement: Supplementary file 12 — Distribution of the polymorphism differentiating sensitive and tolerant isolates. Each point represents the Fstst value calculated per each 10Kb genome window in the genome. Different colors indicate the position of the window in a different genome scaffold (PNG 1.64 MB) [file 572_2025_1191_Fig9_ESM.png]

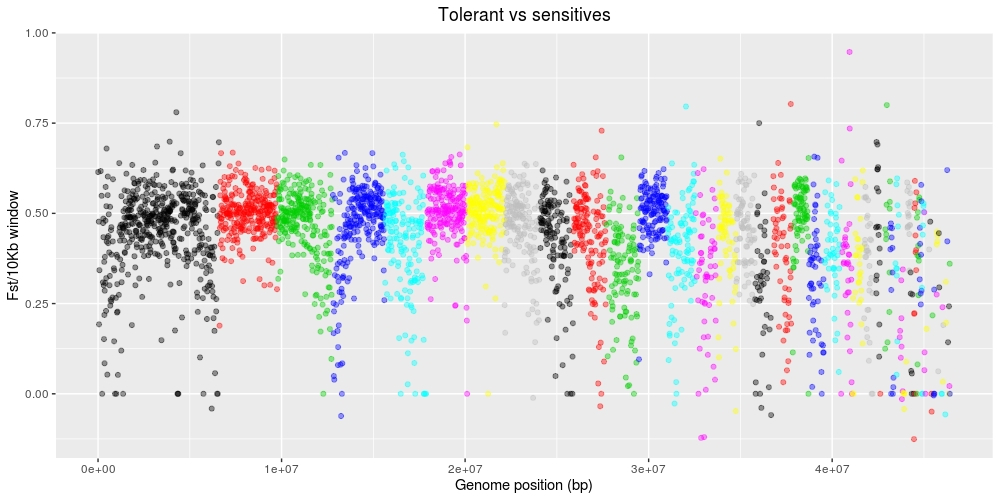

Supplement: Supplementary file 13 — Supplementary file7 (TIFF 1469 KB) [file 572_2025_1191_MOESM7_ESM.tiff]
